# Supplementary material for: Moisture control of tropical cyclones in high-resolution simulations of paleoclimate and future climate
Source: Nat Commun. 2023 Oct 13;14:6426. doi: 10.1038/s41467-023-42033-8 (PMC10575924; doi:10.1038/s41467-023-42033-8)
Supplement: Supplementary file 1 — Supplementary Information [file 41467_2023_42033_MOESM1_ESM.pdf]

Supplementary Information for

Moisture control of tropical cyclones in high-resolution simulations of paleoclimate and future climate

Pavan Harika Raavi[1,2], Jung-Eun Chu[1,3*], Axel Timmermann[1,4], Sun-Seon Lee[1,4], and Kevin J. E. Walsh[5]

[1]Center for Climate Physics, Institute for Basic Science (IBS), Busan, Republic of Korea, 46241
[2]Centre for Climate Research Singapore, Singapore
[3]Low-Carbon and Climate Impact Research Centre, School of Energy and Environment, City University of Hong Kong, Hong Kong, China
[4]Pusan National University, Busan, Republic of Korea, 46241
[5]School of Geography, Earth and Atmospheric Sciences, University of Melbourne, Parkville, Australia

* Corresponding author: Jung-Eun Chu
E-mail: jungeun.chu@cityu.edu.hk

This file includes:

Fig. S1. Annual variations in the difference of the vertical air temperature, specific humidity, and relative humidity profiles between two past climate simulations (Marine Isotope Stages 5e (MIS5e) minus 5d (MIS5d)) across the Northern Hemisphere basins.

Fig. S2. Zonally averaged moist entropy deficit and static stability of the atmosphere across four different model climates (Marine Isotope Stages 5e (MIS5e) and 5d (MIS5d), present-day (PD), and future (2×CO_2)).

Fig. S3. Annual mean Genesis Potential Index (GPI)[1] in the four different model climates and their difference due to orbital and greenhouse gas forcings.

Fig. S4. Genesis Potential Index (GPI)[2] variational component analysis.

Fig. S5. Annual variations in the differences of the vertical air temperature, specific humidity, and relative humidity profiles in the past and future climates.

Fig. S6. Climatological seasonal mean differences of thermodynamical and dynamical conditions in the orbital forcing experiments (summer in each hemisphere).

Fig. S7. Climatological seasonal mean differences of precipitation in the orbital forcing and greenhouse warming experiments.

Fig. S8. A category-4 tropical cyclone (TC) case in MIS5e simulation.

Fig. S9. Simulated tropical cyclone (TC) tracks and annual frequency in global and hemisphere contexts.

Fig. S10. Tropical cyclone (TC) annual cycle from the International Best Track Archive for Climate Stewardship (IBTrACS) and from different climate simulations.

Fig. S11. Tropical cyclone (TC) genesis density differences estimated within a 5°×5° grid box for the TCs detected using Okubo-Weiss-Zeta parameter (OWZP) tracking scheme.

Fig. S12. Same as Figure S6 but for the increased greenhouse gas forcing simulations (2×CO_2 minus PD).

Fig. S13. Tropical cyclone (TC) maximum 10m wind speeds and minimum sea level pressure during the lifetime of the storm detected by traditional tracking scheme[3].

Fig. S14. Monthly sea surface temperature (SST) differences in paleoclimate and future climate simulations.

Fig. S15. Monthly maximum potential intensity (MPI) differences in paleoclimate and future climate simulations.

Table S1: Initial and core thresholds for tracking tropical cyclones (TCs) using Okubo–Weiss–Zeta parameter (OWZP) detection scheme.

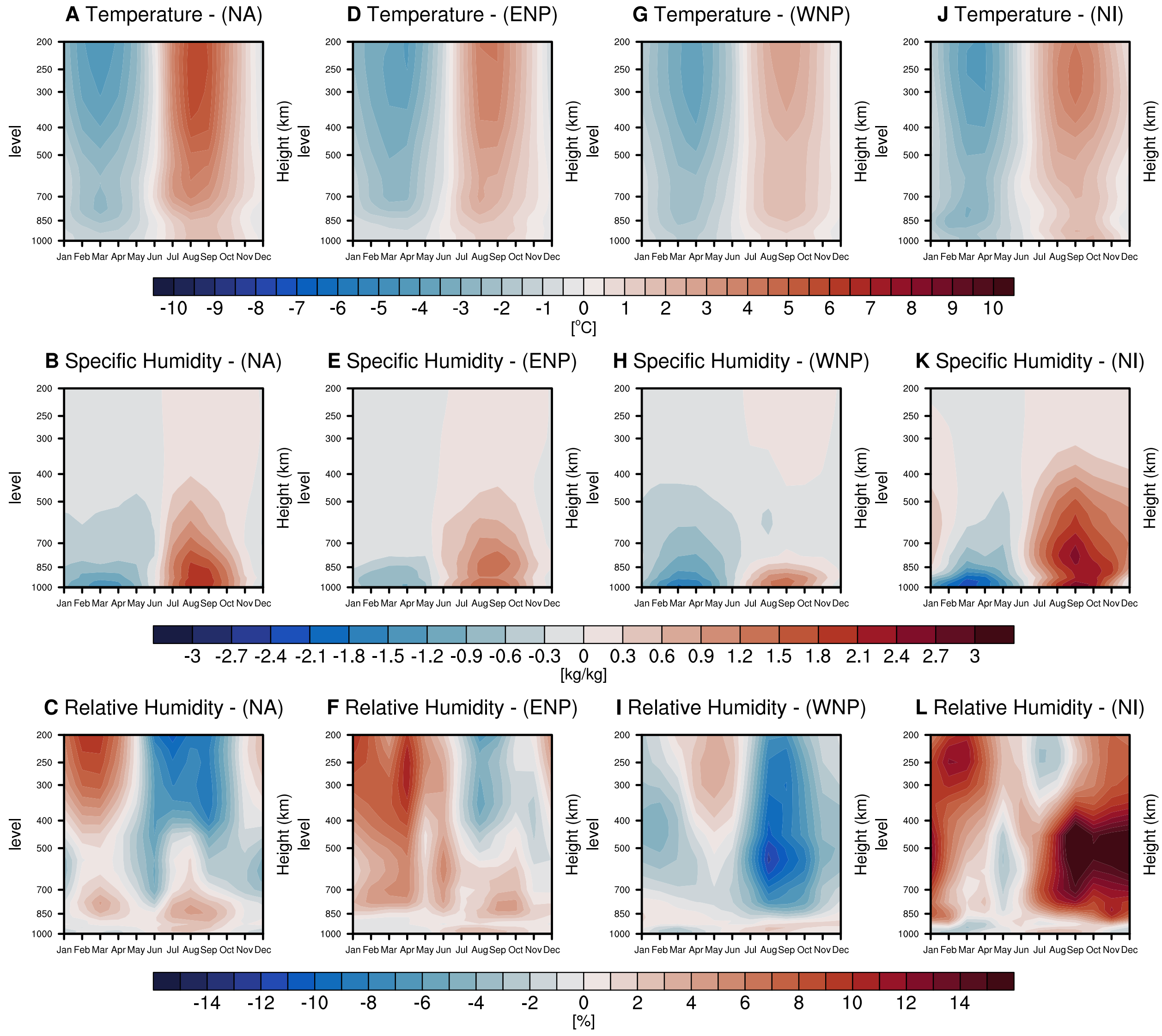

Fig. S1. Annual variations in the difference of the vertical air temperature, specific humidity, and relative humidity profiles between two past climate simulations (Marine Isotope Stages 5e (MIS5e) minus 5d (MIS5d)) across the Northern Hemisphere basins. (A–C) North Atlantic (NA, EQ–30°N; 100°W–0°W), (D–F) Eastern North Pacific (ENP, EQ–30°N; 180°E-100°W), (G–I) Western North Pacific (WNP, EQ–30°N; 100°E–180°E), and (J–L) North Indian Ocean (NI, EQ–30°N; 40°E–100°E).

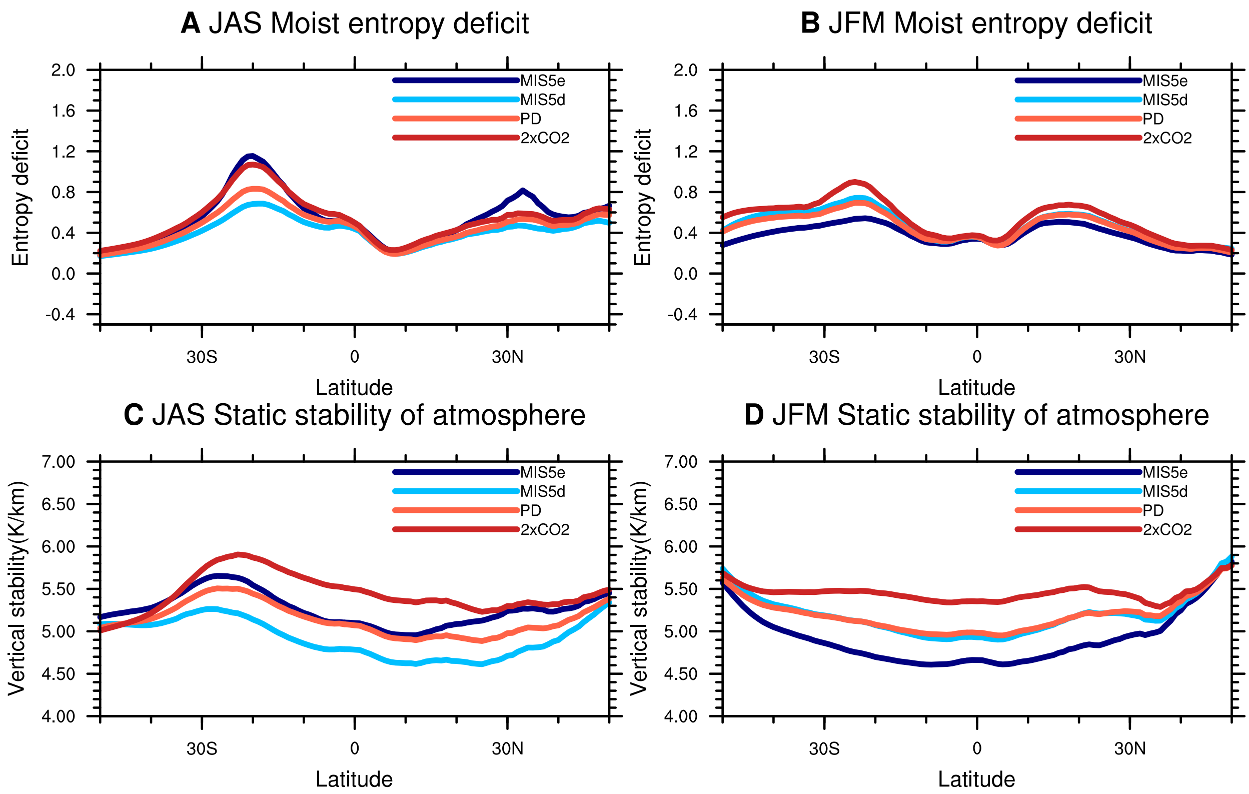

Fig. S2. Zonally averaged moist entropy deficit and static stability of the atmosphere across four different model climates (Marine Isotope Stages 5e (MIS5e) and 5d (MIS5d), present-day (PD), and future (2×CO_2)). (A–B) Moist entropy deficit between the boundary layer and 600 hPa of the atmosphere and (C–D) static stability of the atmosphere (between the 925 and 200 hPa pressure levels) in the tropical cyclone peak seasons (i.e., July to September (JAS) for the Northern Hemisphere summer; January to March (JFM) for the Southern Hemisphere summer).

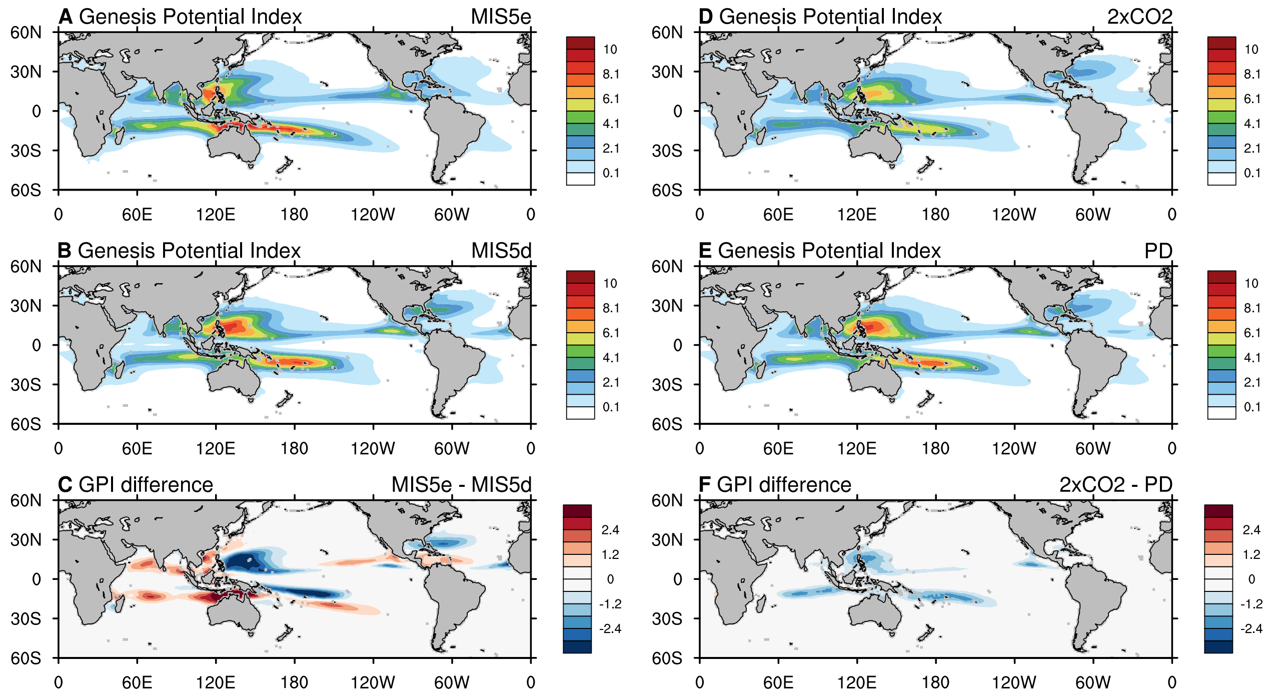

Fig. S3. Annual mean Genesis Potential Index (GPI)[1] in the four different model climates and their difference due to orbital and greenhouse gas forcings. GPI from the past climates (A) Marine Isotope Stages 5e (MIS5e) and (B) 5d (MIS5d), (D) future climate (2×CO_2), and (E) present-day (PD) simulations. Difference in GPI due to (C) orbital forcing (MIS5e minus MIS5d) and (F) greenhouse gas forcing (2×CO_2 minus PD).

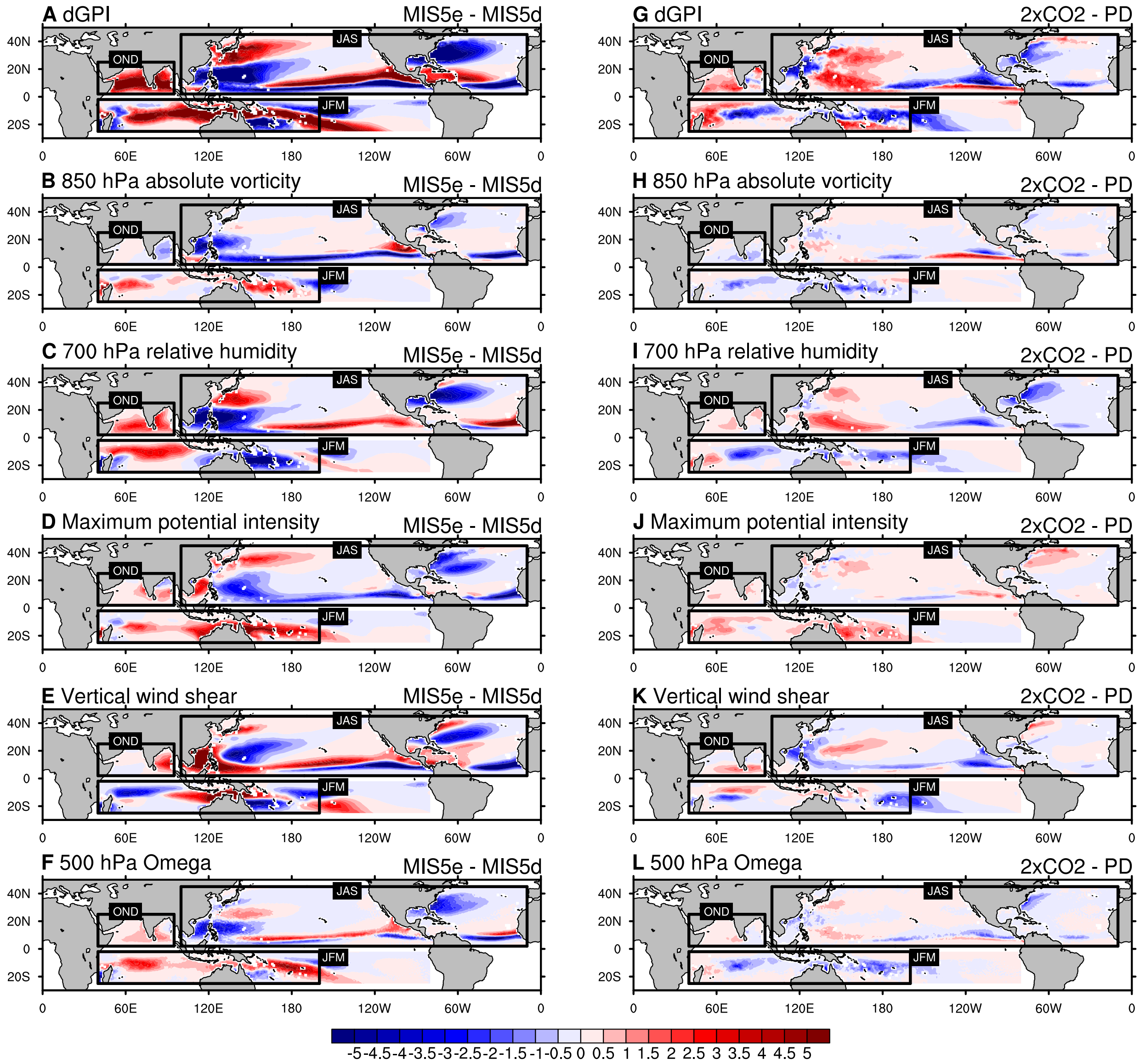

Fig. S4. Genesis Potential Index (GPI)[2] variational component analysis. Changes in GPI and components from (A–F) orbital forcing simulations (MIS5e minus MIS5d) and (G–L) increased CO_2 concentration simulations (2×CO_2 minus PD) for a period of 60 years from each simulation during the tropical cyclone (TC) peaks seasons across different ocean basins (i.e., June to September in the North Pacific and North Atlantic basins; January to March in Southern Hemisphere basins; October to December in the Northern Indian basin).

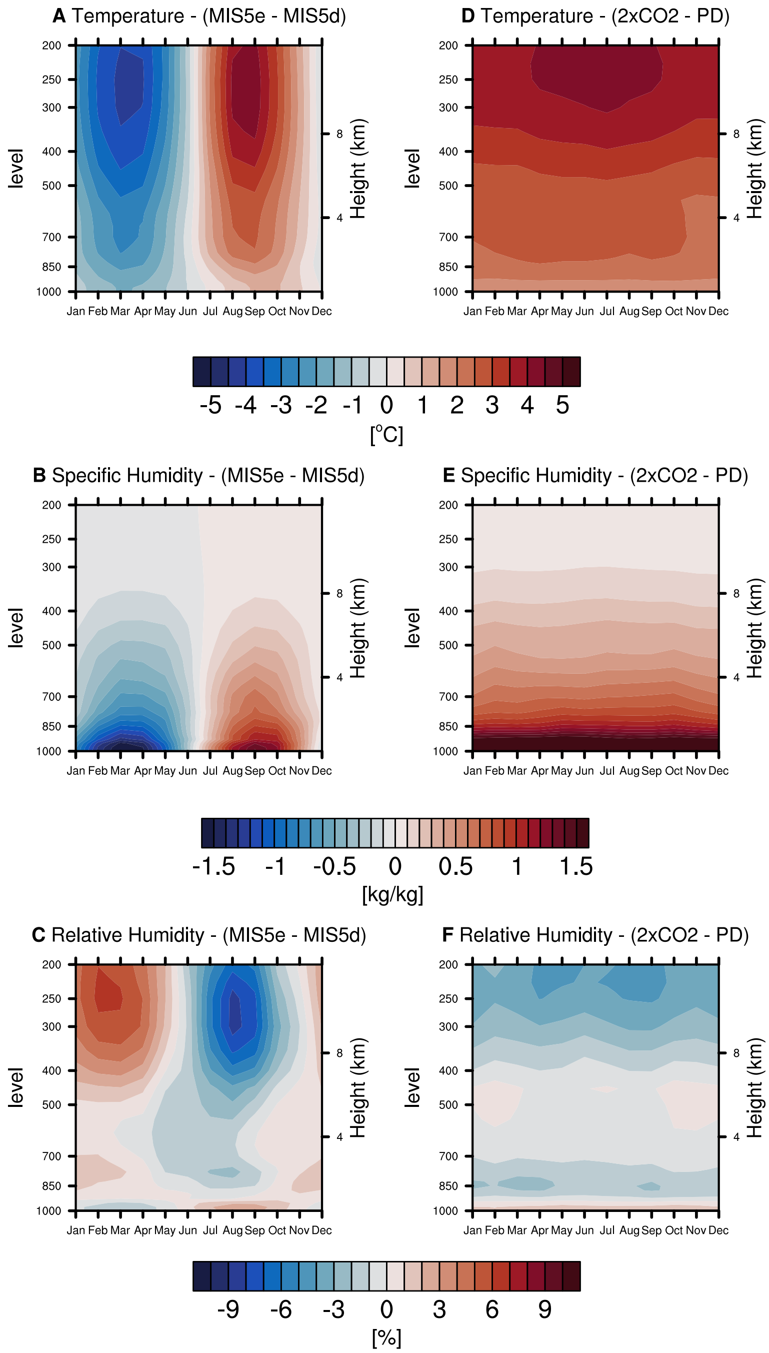

Fig. S5. Annual variations in the differences of the vertical air temperature, specific humidity, and relative humidity profiles in the past and future climates. Differences in area-averaged air temperature, specific humidity, and relative humidity between the orbital forcing simulations (MIS5e minus MIS5d) and greenhouse gases forcing simulations (2×CO_2 minus PD) within a latitude band of 30°S–30°N over the whole longitudes.

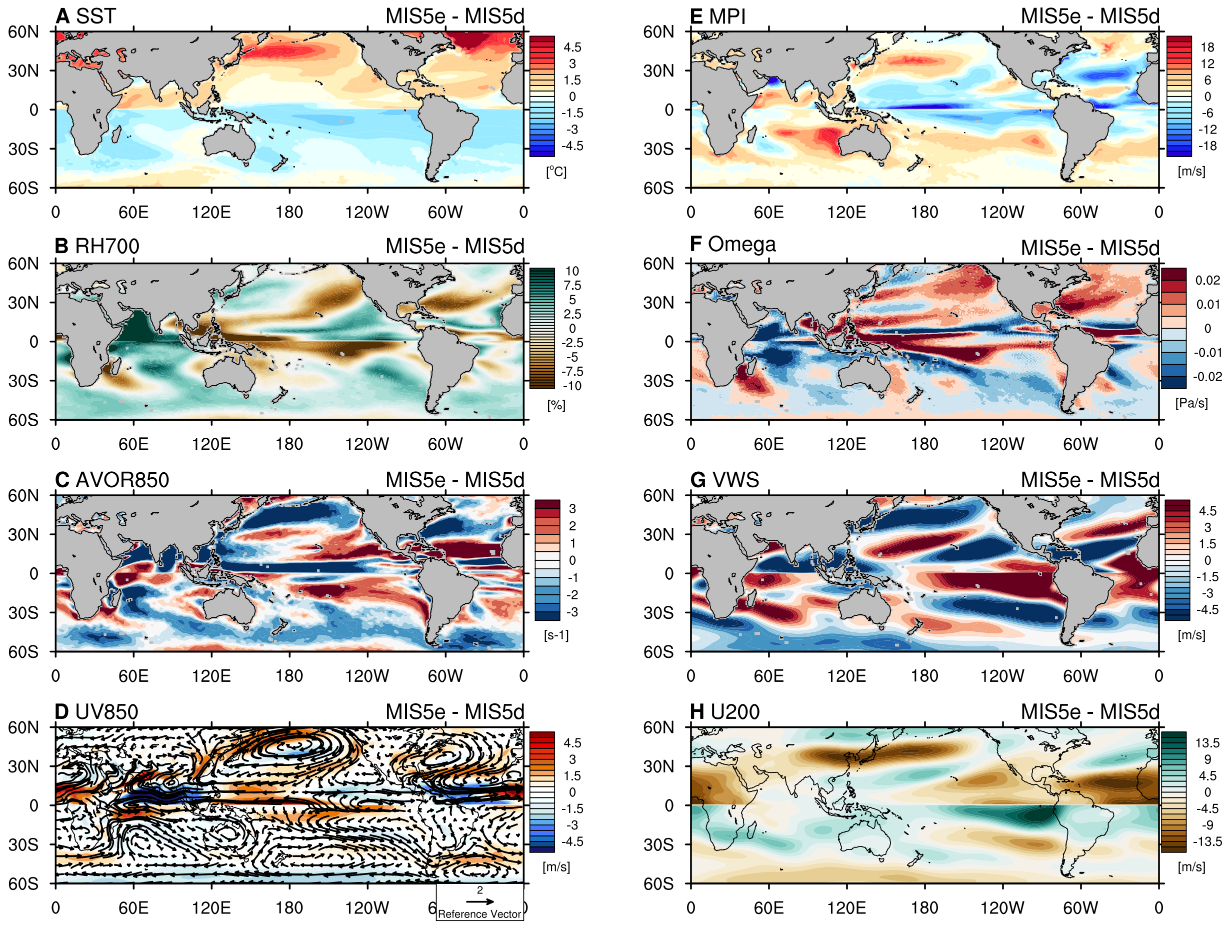

Fig. S6. Climatological seasonal mean differences of thermodynamical and dynamical conditions in the orbital forcing experiments (summer in each hemisphere). Differences in (A) sea surface temperatures (°C), (B) 700 hPa relative humidity (%), (C) 850 hPa absolute vorticity (s^{-1}), (D) 850 hPa winds (m s^{-1}, shading indicates the wind speed, vectors denote the directions), (E) maximum potential intensity, (F) 500 hPa omega (m s^{-1}), (G) vertical wind shear between 850 and 200 hPa levels (m s^{-1}), and (H) 200 hPa zonal wind (m s^{-1}) between orbital forcing simulations (MIS5e minus MIS5d).

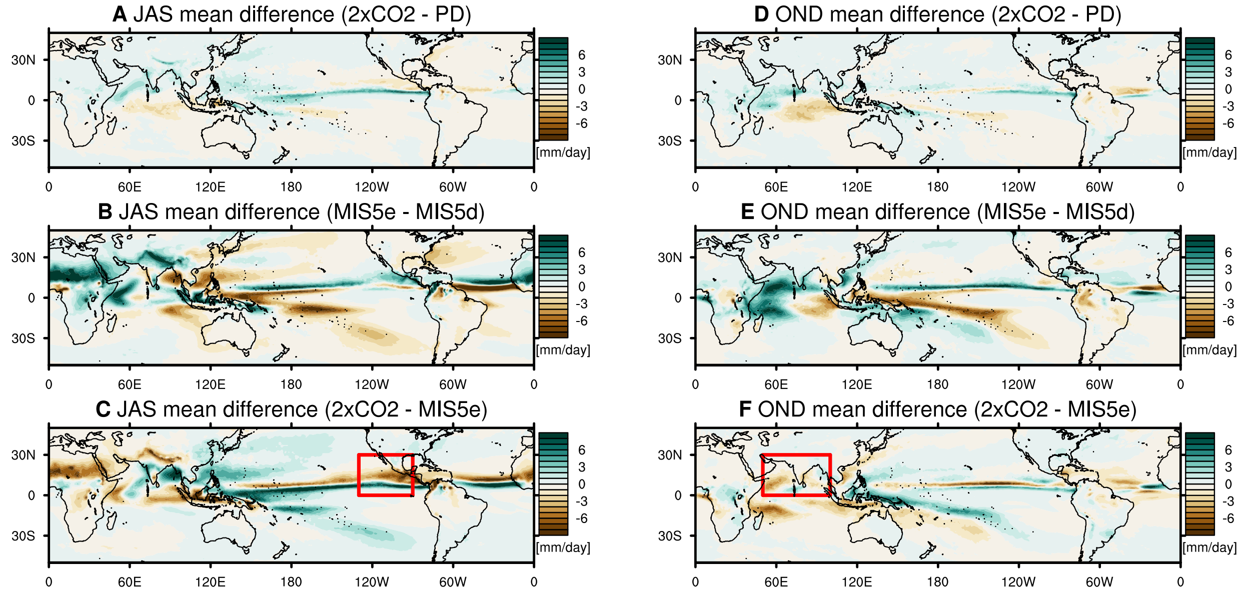

Fig. S7. Climatological seasonal mean differences of precipitation in the orbital forcing and greenhouse warming experiments. (A and D) 2×CO_2 minus PD, (B and E) MIS5e minus MIS5d, and (C and F) 2×CO_2 minus MIS5e for July to September (left panel) and October to December (right panel) seasons. The red box shows the poleward shift of Intertropical Convergence Zone (ITCZ) position in the Eastern North Pacific (left) and Northern Indian Ocean (right) in the MIS5e.

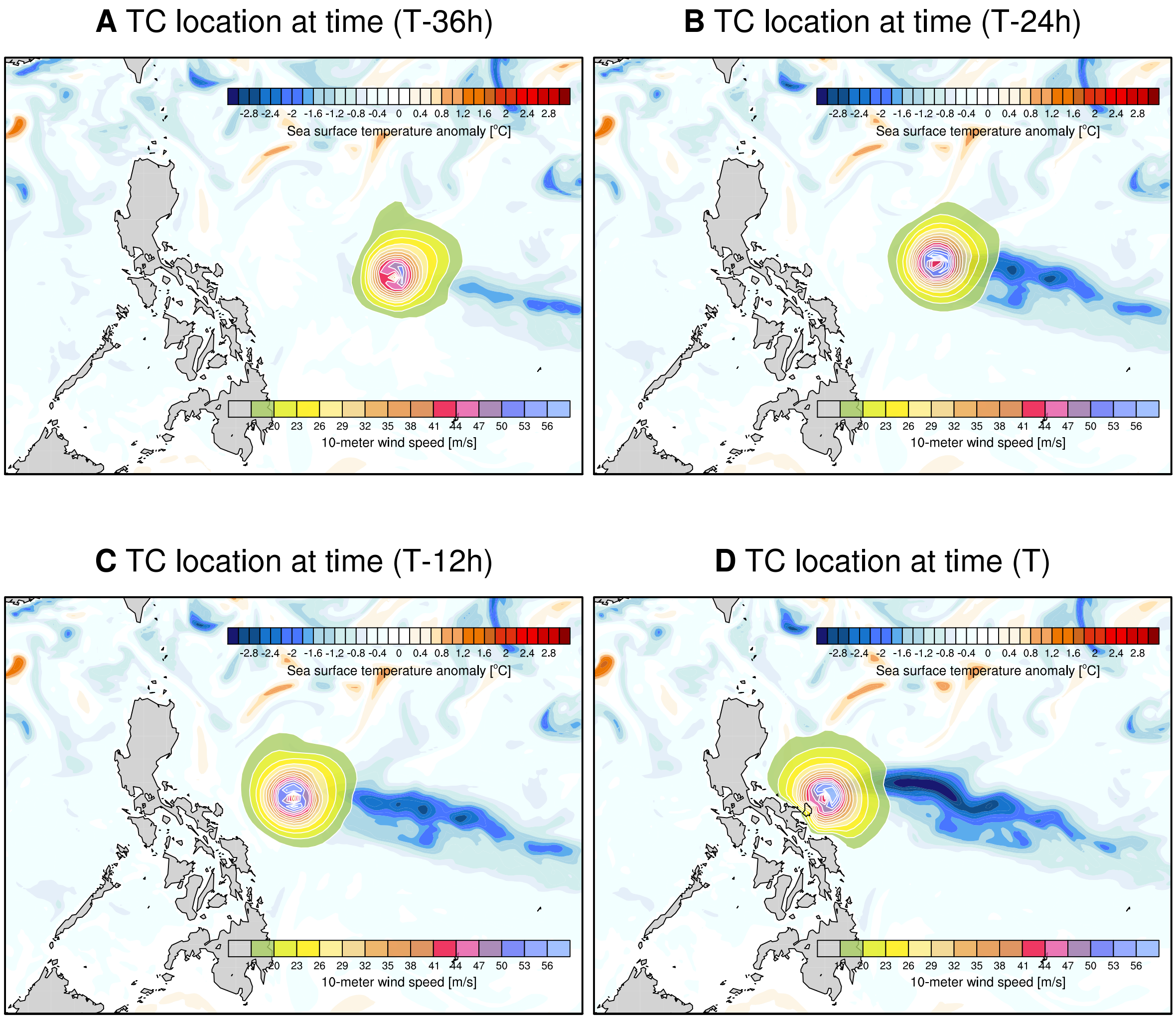

Fig. S8. A category-4 tropical cyclone (TC) case in MIS5e simulation. Sea surface temperature (SST) anomaly and TC surface wind speeds for a simulated TC case in the high-resolution Community Earth System Model (CESM). SST anomaly is calculated by subtracting the previous 14-day averaged SST.

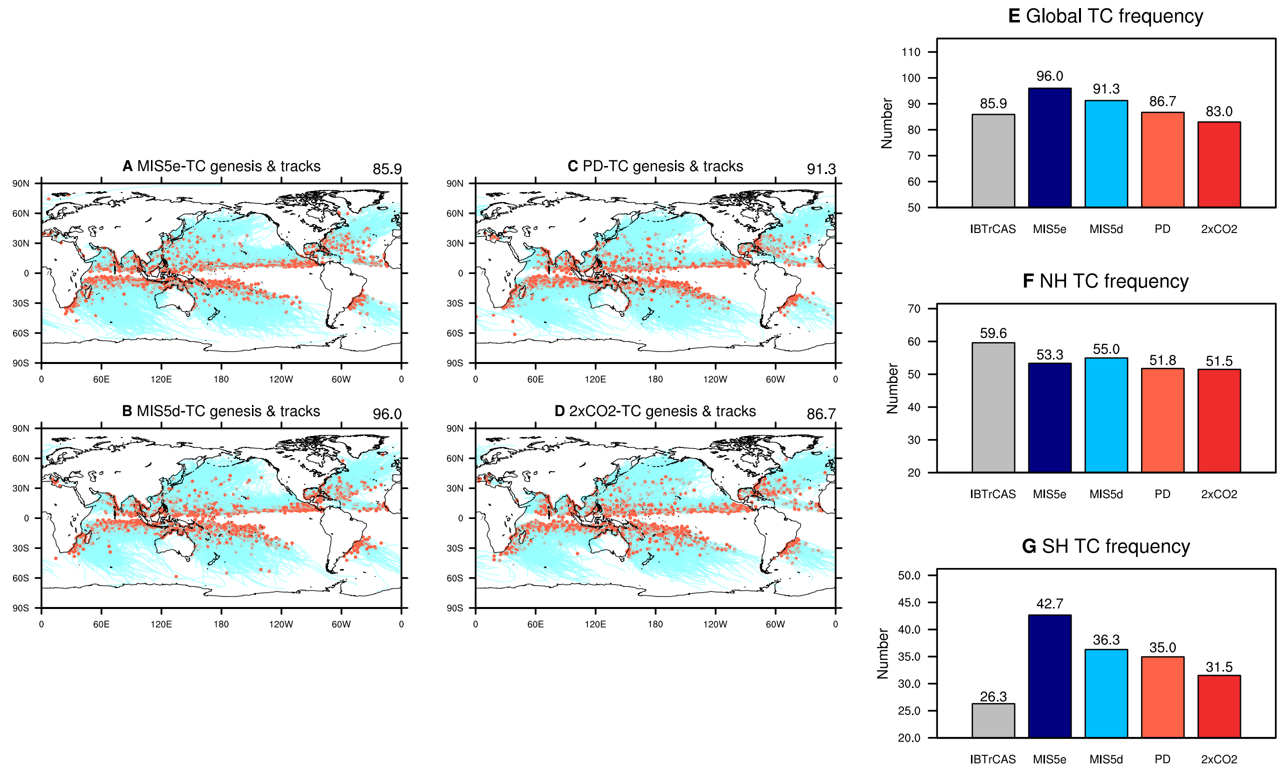

Fig. S9. Simulated tropical cyclone (TC) tracks and annual frequency in global and hemisphere contexts. TC genesis and tracks detected using the traditional TC tracking scheme[3] from the (A) MIS5e, (B) MIS5d, (C) PD, and (D) 2×CO_2 simulations. Annual TC frequency from the International Best Track Archive for Climate Stewardship (IBTrACS) and different model climates for (E) global, (F) Northern Hemisphere (NH), and (G) Southern Hemisphere (SH) domains.

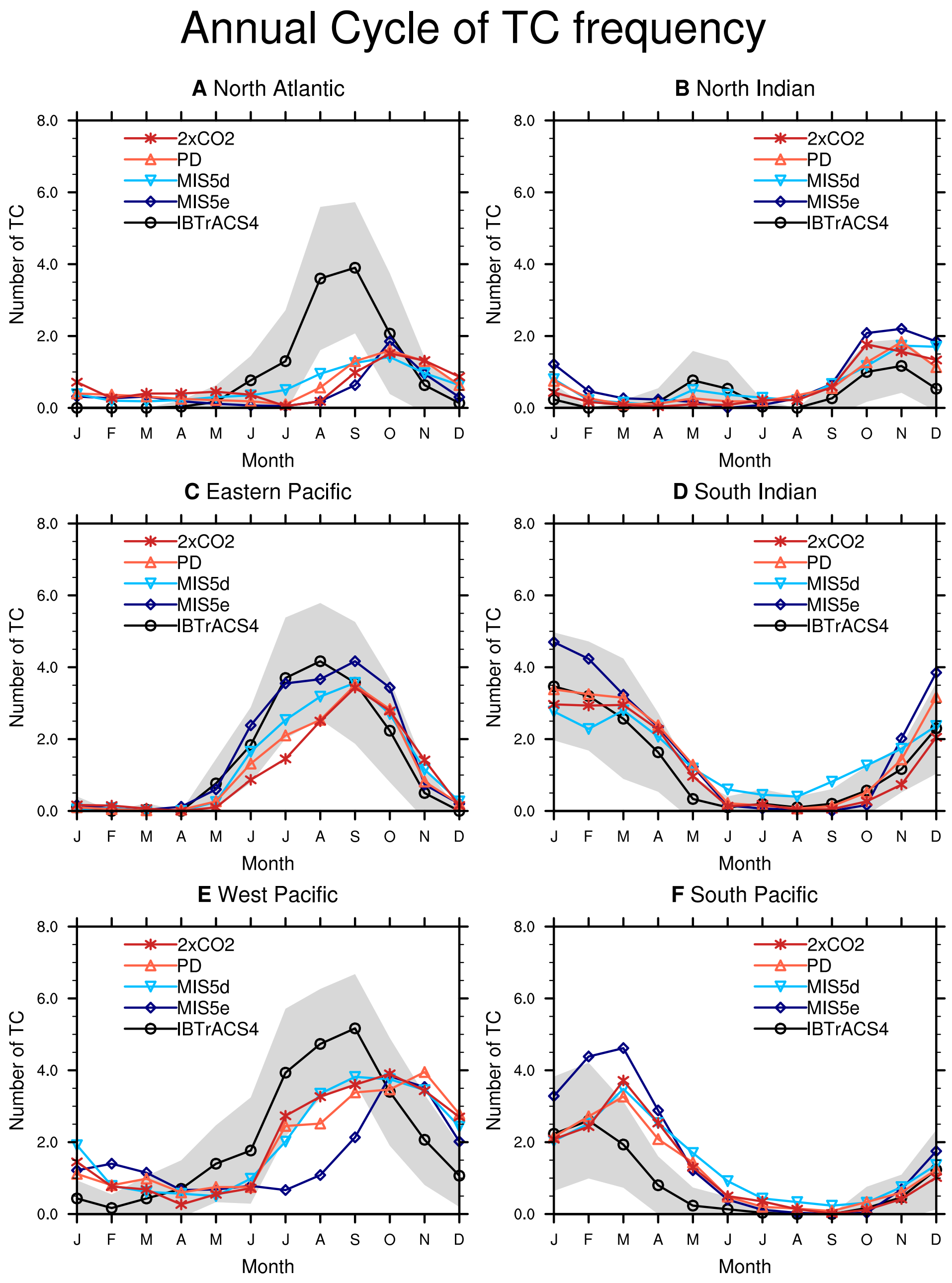

Fig. S10. Tropical cyclone (TC) annual cycle from the International Best Track Archive for Climate Stewardship (IBTrACS) and from different climate simulations. Annual variations in the frequency of TC genesis across global ocean basins in the IBTrACS observation, paleoclimate (MIS5e and MIS5d), present-day (PD), and future climate (2×CO_2). Gray shading indicates the interannual variability of TC frequency in the IBTrACS.

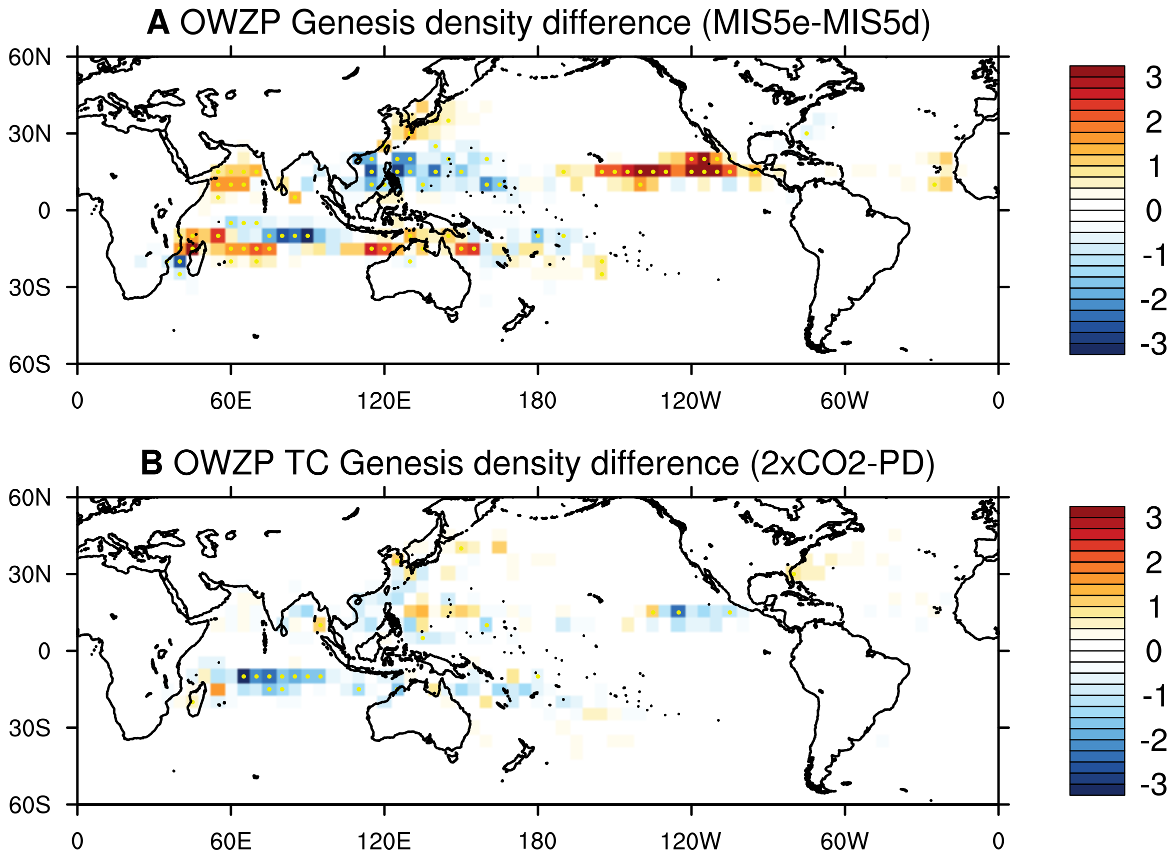

Fig. S11. Tropical cyclone (TC) genesis density differences estimated within a 5°×5° grid box for the TCs detected using Okubo-Weiss-Zeta parameter (OWZP) tracking scheme. (A) Orbital forcing experiments (MIS5e minus MIS5d) and (B) greenhouse gas forcing experiments (2×CO_2 minus PD). Yellow dots indicate the statistically significant (95%) difference measured using a two-sample *t*-test.

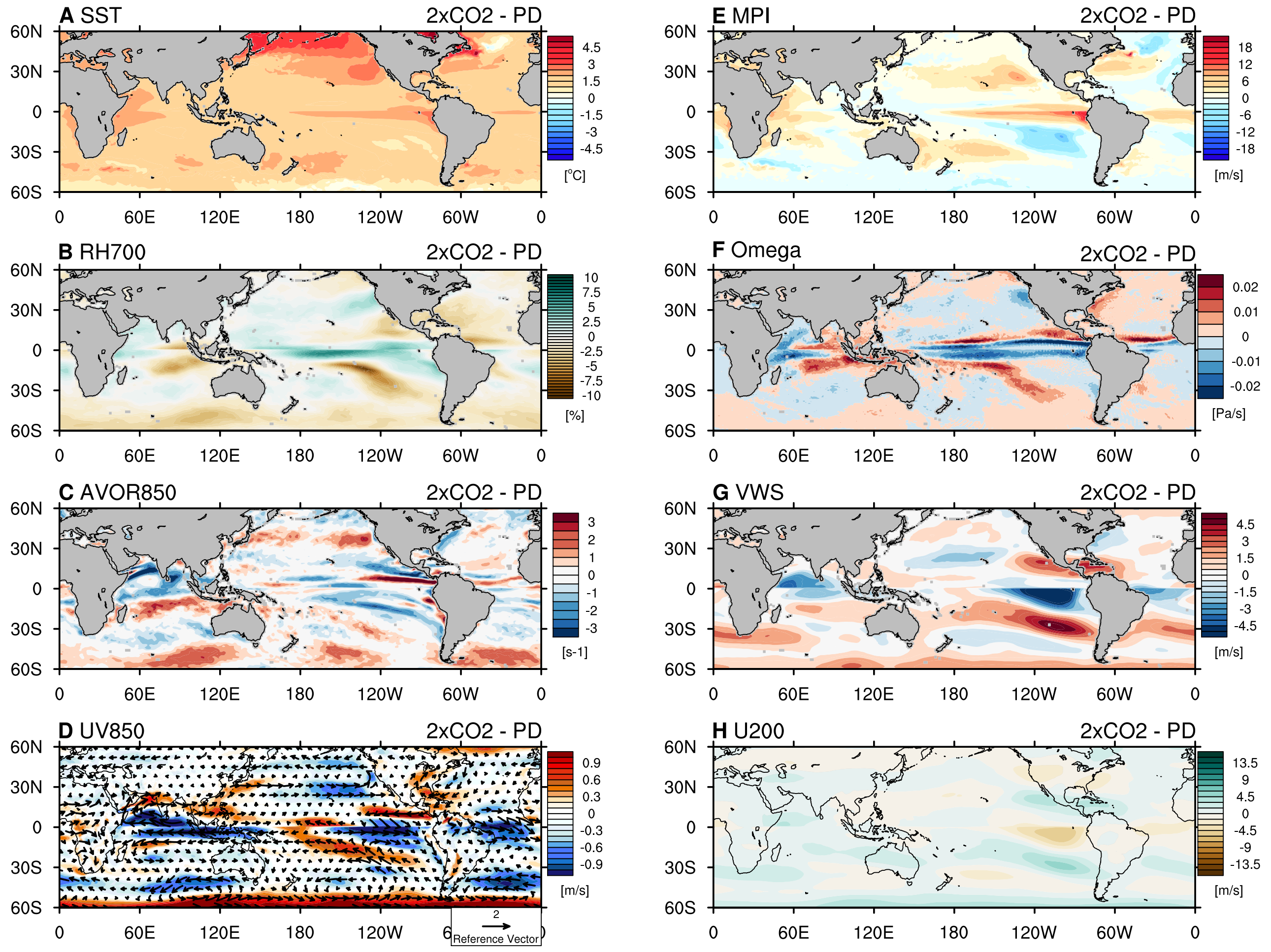

Fig. S12. Same as Figure S6 but for the increased greenhouse gas forcing simulations (2×CO_2 minus PD). Note that color bars in (D) 850 hPa winds and (H) 200 hPa zonal wind have different magnitudes from that in Fig. S6.

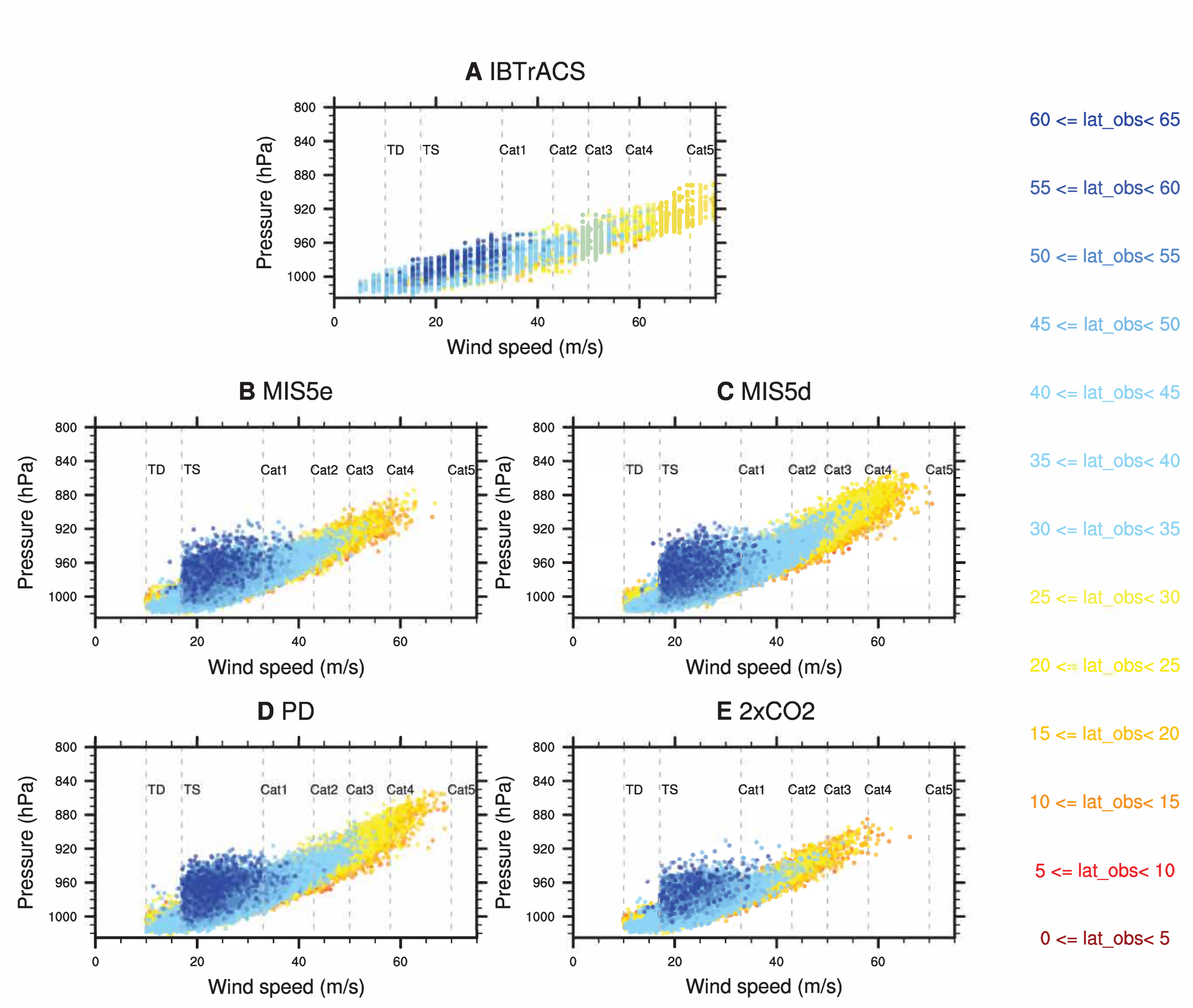

Fig. S13. Tropical cyclone (TC) maximum 10m wind speeds and minimum sea level pressure during the lifetime of the storm detected by traditional tracking scheme[3]. (A) International Best Track Archive for Climate Stewardship (IBTrACS) observation and (B) MIS5e, (C) MIS5d, (D) present-day (PD), and (E) 2×CO_2 simulations. Different colors represent different latitudes, with red colors indicating equatorial regions and blue colors representing higher latitudes.

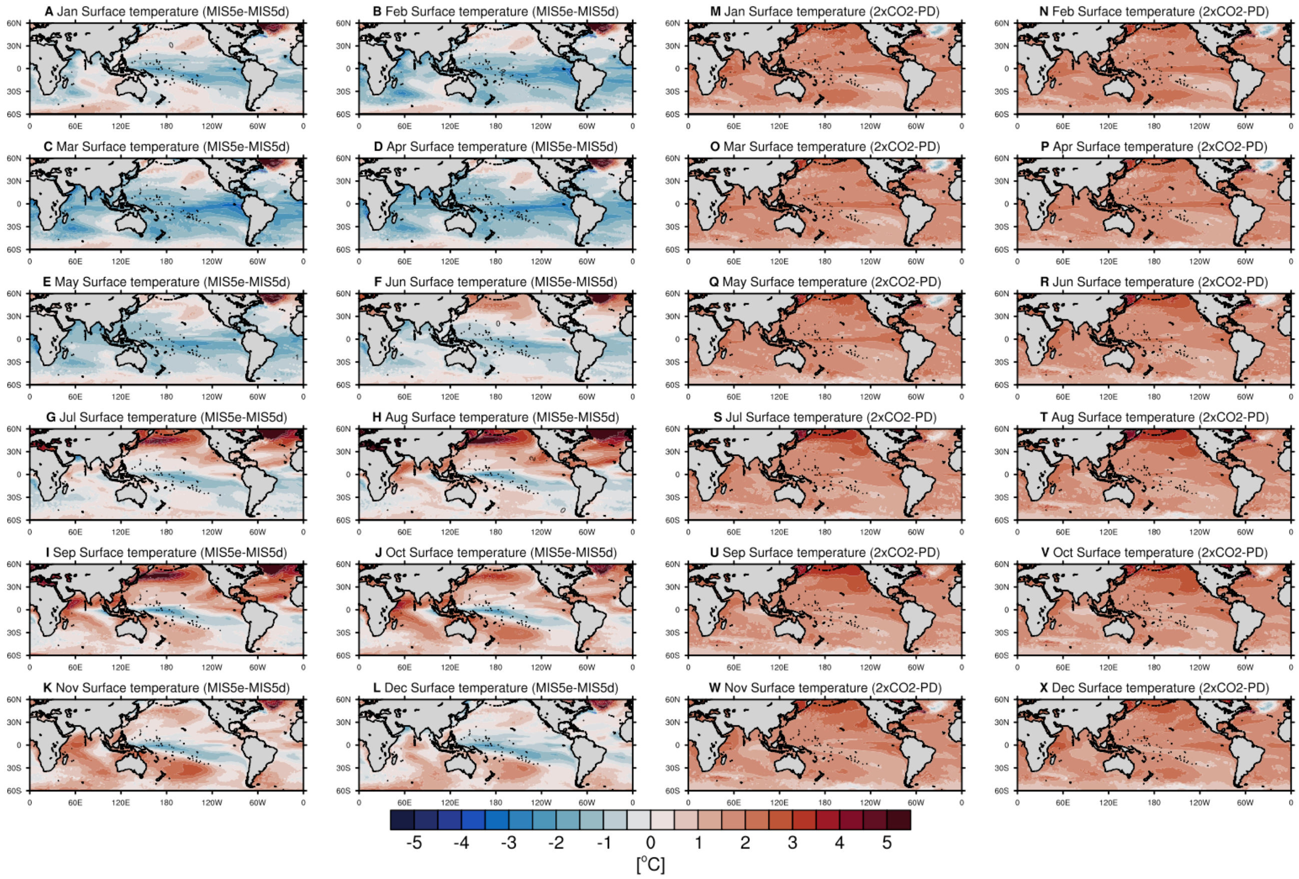

Fig. S14. Monthly sea surface temperature (SST) differences in paleoclimate and future climate simulations. January to December SST difference between (A–L) MIS5e and MIS5d, and (M–X) 2×CO_2 and PD.

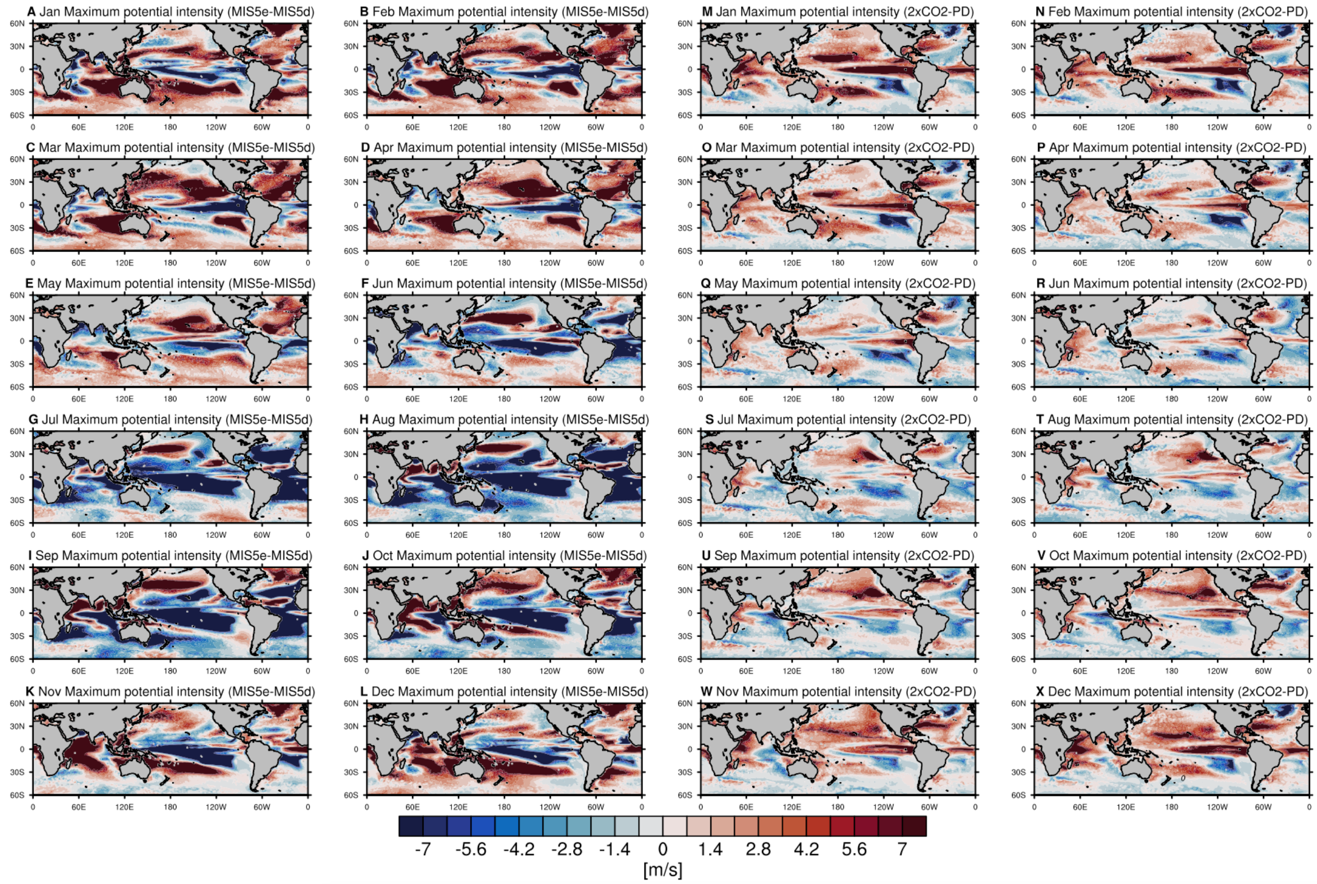

Fig. S15. Monthly maximum potential intensity (MPI) differences in paleoclimate and future climate simulations. January to December MPI difference between (A–L) MIS5e and MIS5d, and (M–X) 2×CO_2 and PD.

Table S1: Initial and core thresholds for tracking tropical cyclones (TCs) using Okubo–Weiss–Zeta parameter (OWZP) detection scheme.

Criterion	OWZ_{850}	OWZ_{500}	RH_{950}	RH_{700}	$VWS_{850\text{-}200}$	SH_{950}
**Initial**	$50 \times 10^{-6} s^{-1}$	$40 \times 10^{-6} s^{-1}$	70%	50%	$25 ms^{-1}$	$10\ g\ kg^{-1}$
**Core**	$60 \times 10^{-6} s^{-1}$	$50 \times 10^{-6} s^{-1}$	85%	70%	$12.5 ms^{-1}$	$14\ g\ kg^{-1}$

SH denotes specific humidity, RH denotes relative humidity, and VWS denotes vertical wind shear. Subscript denotes the pressure levels.

The equation for the calculation of low deformation vorticity (OWZ) given below:

$$OWZ = \max\left(OW_{norm,}\, 0\right) \times \left(\xi + f\right) \times sign\left(f\right)$$

$$OW_{norm} = \frac{\xi^2 - (E^2 + F^2)}{\xi^2}$$

$$E = \left[\frac{du}{dx}\right] - \left[\frac{dv}{dy}\right] \qquad F = \left[\frac{dv}{dx}\right] + \left[\frac{du}{dy}\right]$$

Here OW_{norm} is the normalized Okubo-Weiss parameter, ξ is the relative vorticity, f is the planetary vorticity, $\zeta + f$ is the absolute vorticity, while E and F represent the stretching and shearing deformation, respectively.

Supplementary References

1 Korty, R. L., Camargo, S. J. & Galewsky, J. Variations in Tropical Cyclone Genesis Factors in Simulations of the Holocene Epoch. *Journal of Climate* **25**, 8196-8211 (2012). https://doi.org/10.1175/Jcli-D-12-00033.1

2 Murakami, H. & Wang, B. Future Change of North Atlantic Tropical Cyclone Tracks: Projection by a 20-km-Mesh Global Atmospheric Model. *Journal of Climate* **23**, 2699-2721 (2010). https://doi.org/10.1175/2010jcli3338.1

3 Chu, J. E. *et al.* Reduced tropical cyclone densities and ocean effects due to anthropogenic greenhouse warming. *Science Advances* **6** (2020). https://doi.org/ARTN eabd510910.1126/sciadv.abd5109
